# An alternative microRNA-mediated post-transcriptional regulation of GADD45A by p53 in human non-small-cell lung cancer cells

**DOI:** 10.1038/s41598-017-07332-3

**Published:** 2017-08-02

**Authors:** Jie Li, Jie Dong, Shaohua Li, Wei Xia, Xueting Su, Xingliang Qin, Ying Chen, Hongmei Ding, Hui Li, Aixue Huang, Chenjun Bai, Tongnan Hu, Chenglong Wang, Bingfeng Chu, Ningsheng Shao

**Affiliations:** 1 Department of Biochemistry and Molecular Biology, Institute of Basic Medical Sciences, Beijing, 100850 China; 2 Department of Stomatology, Chinese PLA General Hospital, Beijing, 100853 China

## Abstract

GADD45A (growth arrest and DNA damage inducible alpha), a stress response gene induced by genotoxic and nongenotoxic stresses, is implicated in various key processes, including the control of cell cycle checkpoints and DNA repair. The expression of GADD45A is directly regulated by numerous transcription factors, with p53 being the most representative. Moreover, post-transcriptional regulation also plays a role in GADD45A expression. However, little is known about the regulatory effects of microRNAs (miRNAs) on GADD45A expression. As a potential tumour suppressor, miR-138 has pleiotropic biological functions in various cancers. We have previously reported p53-mediated activation of miR-138 in human non-small-cell lung cancer (NSCLC) cells. In this study, we found that miR-138 specifically targeted AGO2, which affects the stability and maturation of miR-130b. Decreased expression of miR-130b promoted the expression of GADD45A and resulted in the G2/M phase arrest and proliferation inhibition in human NSCLC cells. Our results suggested that p53 could alternatively upregulate GADD45A in human NSCLC cells through a post-transcriptional pathway in which miR-138 is involved.

## Introduction

As a transcription regulator, p53 plays a prominent role in cellular responses to stress signals, such as DNA damage, oncogene activation, and hypoxia. It does this by regulating the expression and coordinating the activity of multiple effectors, which consequently modulate key cellular processes such as apoptosis, cellular proliferation, and autophagy^1–3^. Inactivation of p53 function is the most common event in human cancer, leading to a dysregulated cell cycle, genomic instability, resistance to stress signals, and ultimately cancer development^4–6^.

*Gadd45a* was the first stress gene discovered to be that was transcriptionally regulated by p53^7^. The *Gadd45a* gene encodes an acidic protein of approximately 18 kDa that is induced by both genotoxic stresses (e.g., ultraviolet radiation (UVR), ionizing radiation (IR), and Adriamycin) and nongenotoxic stresses (e.g., apoptotic and/or growth-inhibitory cytokines, serum starvation, and endoplasmic reticulum stress agents)^8, 9^. GADD45A protein plays an important role in maintenance of genomic stability, cell cycle control, apoptosis, and DNA repair. The expression of *GADD45A* is upregulated by IR in a p53-dependent manner, while non-IR factors such as UVR and serum starvation activate *GADD45A* in a p53-independent manner via BRCA1, OCT1, NF-YA and other proteins^10–13^. Further studies have indicated two main mechanisms of p53 induction of *GADD45A* expression. One is the direct transcriptional regulation by p53 via binding to a conserved site within the third intron segment of *GADD45A* ^14^, and the other is the indirect regulation of p53 via transcriptional regulation of other proteins, such as WT1 protein^15^.

MicroRNAs (miRNAs) are a class of endogenous small non-coding RNAs of approximately 20–25 nucleotides in length that function as post-transcriptional gene expression regulators, either through translational repression or by mRNA degradation. miRNAs play vital roles in a wide variety of cell processes by regulating crucial genes^16, 17^. Dysregulation of miRNA expression is associated with aberrant gene expression and is involved in pathological conditions of various diseases, such as cancers^18–20^. Recently, p53 was found to regulate miRNAs by facilitating transcription or modulating the biogenesis of miRNAs, and miRNAs were identified as key elements in the p53 network^21, 22^.

Our previous study indicated that p53 activated miR-138 transcription in human NSCLC cells^23^. miR-138 has a variety of biological functions due to its ability to act on different target genes in various cells and tissues^24–26^. Herein, we conducted a study of the miR-138 regulatory pathway initiated by p53 to broaden our knowledge of the p53 network. We found that miR-138 specifically targeted AGO2, which affected the stability and maturation of miR-130b. Our subsequent searching of miR130b targets in the p53 signalling pathway identified GADD45A, which affect cell cycle and proliferation in human NSCLC cells.

## Results

### p53 downregulates AGO2 expression by a miR-138-mediated pathway

**Figure 1:**
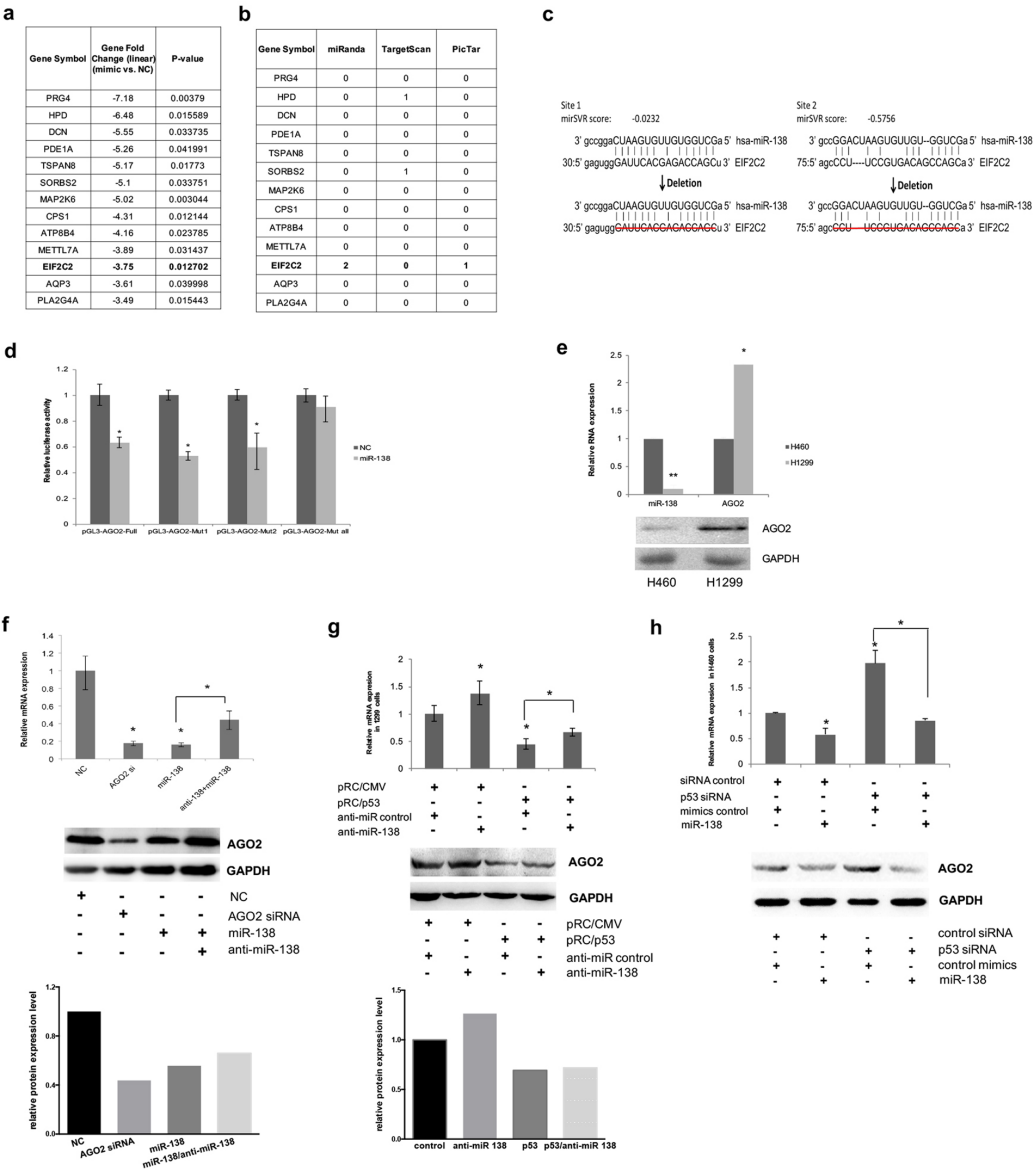
AGO2 (EIF2C2) is a miR-138 target in human NSCLC cells. miR-138 targets identified using a microarray analysis and bioinformatics in H1299 cells (**a**,**b**). ^a^Only the top thirteen highest-ranking downregulated genes are listed (for more details, see GEO: GSE69482). (**c**) Schematic representation of miR-138 targets in the 3′ UTR of human *AGO2* (top). The positions of miR-138 binding sites correspond to the locations in GenBank accession NM_001164623. The artificial mutant 3′ UTR without the miR-138 binding sites is shown in the bottom image. (**d**) The full length 3′ UTR of *AGO2*, containing two miR-138 binding sites, was cloned downstream of a firefly luciferase gene, and the plasmid was named pGL3-AGO2-Full. *AGO2* 3′ UTR-containing reporter plasmids with either or both miR-138 mutant binding sites were named pGL3-AGO2-Mut1, pGL3-AGO2-Mut2 and pGL3-AGO2-Mut-all, respectively. Dual-luciferase reporter assays were performed to test the interaction between miR-138 and the predicted wild-type *AGO2* 3′ UTR targeting sequence (pGL3-AGO2-Full) and the mutated targeting sequences (pGL3-AGO2-Mut1, pGL3-AGO2-Mut2, and pGL3-AGO2-Mut-all). A CMV-driven Renilla luciferase construct was co-transfected as a normalization control for firefly luciferase activity. The columns represent the mean normalized relative luciferase activity (RLU) from three independent experiments, with 95% confidence intervals. *P < 0.05 *vs*. NC by rank-sum test. (**e**) *AGO2* mRNA and miR-138 were quantified using real-time PCR (top), and AGO2 protein expression was detected by western blotting in H460 and H1299 cells (bottom). *P < 0.05 and **P < 0.01 vs. NC by rank-sum test. Full-length blots are in Supplementary information. (**f**) *AGO2* mRNA was quantified using real-time PCR (top), and the expression of AGO2 protein was analyzed by western blotting (bottom) in H1299 cells transfected with miR-138, anti-miR-138﻿, ﻿AGO2 siRNA, and negative control. *P < 0.05 *vs*. NC by LSD test. Full-length blots are in Supplementary information. (**g**) *AGO2* mRNA was quantified using real-time PCR (top), the AGO2 protein was analyzed by western blotting and the immunoblots were quantified (bottom) in H1299 cells transfected with p53 expression plasmid (pRC/p53) or pRC/CMV control plasmid. *P < 0.05 by LSD test. Full-length blots are in Supplementary information. (**h**) *AGO2* mRNA was quantified using real-time PCR (top), the AGO2 protein was analyzed by western blotting, and the immunoblots were quantified (bottom) in H460 cells transfected with p53 siRNA or negative control siRNA. *P < 0.05 by LSD test. Full-length blots are in Supplementary information.

A pathway enrichment analysis using miRWalk2.0 database showed that miR-138 was involved in multiple signaling pathway in NSCLC cells (Supplementary Fig. S1a). To identify the target genes of miR-138 in human NSCLC cells, two microarrays with total mRNA from H1299 cells (with a p53-null background) with or without overexpression of miR-138 were analyzed for differential gene expression profiling (GEO: GSE69482) (Fig. 1a). Interestingly, among the top 13 significantly downregulated genes, only the 3′ UTR of *HPD*, *SORBS2* and *EIF2C2* (*AGO2*) were predicted to have target sites for miR-138 using bioinformatics software (miRanda 2010, TargetScan or PicTar), and *AGO2* was the only target gene of miR-138 that was predicted by both miRanda and PicTar (Fig. 1b).

To confirm the direct regulation of *AGO2* by miR-138, we performed a luciferase reporter assay using a series of luciferase reporter constructs carrying the full length *AGO2* mRNA 3′ UTR (879 nt, NM_012154.3) (pGL3-AGO2-Full) with two predicted miRNA-138 target sites (Fig. 1c) or mutants (pGL3-AGO2-Mut1, pGL3-AGO2-Mut2 and pGL3-AGO2-Mut all) in H1299 cells. We observed that miR-138 significantly reduced the luciferase activity of the reporter vectors containing either or both predicted target sites, but had little effect on the control group carrying neither target site (Fig. 1d). The results indicated that miR-138 directly acted on both of the predicted target sites in the *AGO2* 3′ UTR. Further results showed that both the mRNA and protein expression levels of AGO2 were negatively correlated with the endogenous miR-138 level in both the p53-null cell line H1299 and the p53 wild-type cell line H460 (Fig. 1e). When miR-138 was overexpressed in H1299 cells, the expression of *AGO2* was decreased as expected, and addition of a miR-138 inhibitor (anti-miR-138) restored the *AGO2* expression level (Fig. 1f). The results supported the notion that miR-138 specifically targeted AGO2 in the tested NSCLC cell lines.

Because miR-138 specifically targets *AGO2* and we previously confirmed that p53 could activate the expression of miR-138^23^, an experiment to examine p53 regulation of AGO2 was performed. Forced expression of p53 downregulated AGO2 expression at the mRNA and protein levels in p53-deficient H1299 cells, and this downregulation was partially blocked by simultaneous inhibition of miR-138 function using a miR-138 inhibitor at the mRNA level (Fig. 1g). While miR-138 inhibitor increased AGO2 expression by functional inhibition of endogenous base-level miR-138, the effect is undetectable in p53 over-expression cohorts at protein level (Fig. 1g). The main reason that results in the partial restoration at AGO2 mRNA level and undetectable restoration at protein level by the miR-138 inhibitor, as we suspect, was that miR-138 was dramatically increased by over-expression of p53 and exceeded the effective inhibition concentration of exogenous miR-138 inhibitor. While silencing the expression of p53 with siRNA in H460 cells (with p53-wild type background) upregulated AGO2 expression both at the mRNA and protein levels, the upregulation was inhibited by a miR-138 mimic (Fig. 1h). We thus concluded that miR-138 mediated p53 downregulation of AGO2.

### Decreased AGO2 induced by miR-138 affects miR-130b abundance

**Figure 2:**
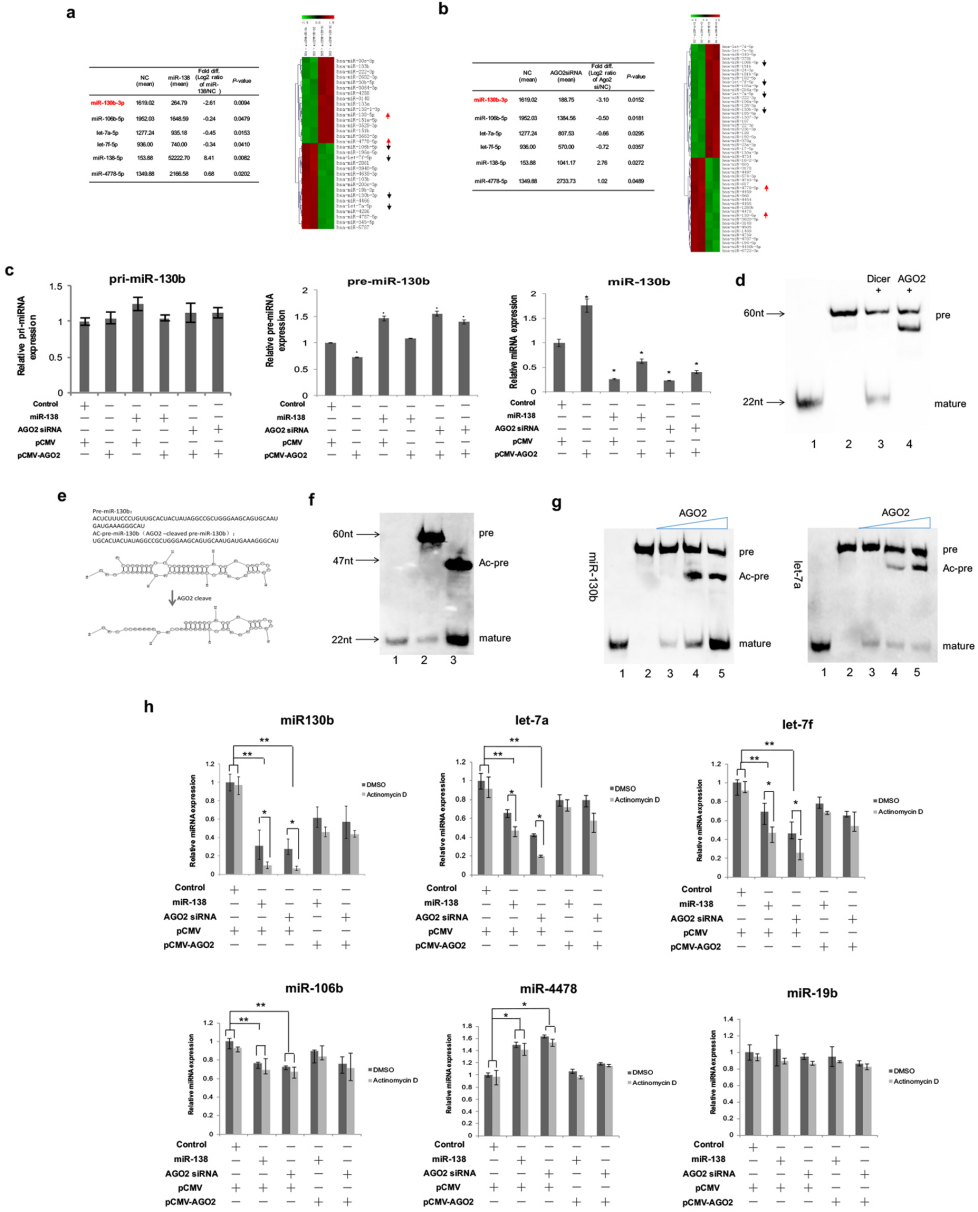
AGO2 affects miR-130b abundance in human NSCLC cells. The miRNAs in H1299 cells that were significantly changed in both the miR-138 and AGO2 siRNA groups *vs*. the negative control (top) (**a**,**b**). The filtered miRNA array data were subjected to unsupervised hierarchical clustering analysis. Upregulated and downregulated miRNAs are represented by red and green colours, respectively, with the common miRNAs marked with red and black arrows, respectively (bottom). (**c**) The effects of AGO2 on the biogenesis of miR-130b. Quantitative RT-PCR assays were performed to examine the effects of AGO2 overexpression, the miR-138 mimic or AGO2 siRNA transfection on miR-130b processing. GAPDH or U6 served as an internal control. *P < 0.05 *vs*. NC by one-way ANOVA test. (**d**) Processing of pre-miR-130b by recombinant Dicer and AGO2 proteins *in vitro*. Briefly, 20 pmol of pre-miR-130b was incubated with 2 U of Dicer or 200 ng of Ago2 for 4 h. The processed products were detected using northern blotting with a biotin-labelled anti-miR-130b probe. Lane 1: 22 nt biotin-ssRNA; 2: pre-miR-130b; 3: pre-miR-130b with Dicer; 4: pre-miR-130b with AGO2. Full-length blots are in Supplementary information. (**e**) The sequence and the predicted secondary structure of Ac-pre-miR-130b. The most abundant sequence and secondary structure (http://rna.urmc.rochester.edu/RNAstructureWeb/Servers) are illustrated. (**f**) Dicer cleavage of miR-130b precursors *in vitro*. Briefly, 20 pmol of miR-130b precursors were incubated with 2 U of Dicer *in vitro* for 4 h and then a northern blot analysis was performed as in **c**. Lane 1: 22 nt biotin-ssRNA; 2: pre-miR-130b; 3: Ac-pre-miR-130b. Full-length blots are in Supplementary information. (**g**) AGO2 catalysis promotes the maturation of miR-130b. Pre-miR-130b was incubated with Dicer (2 U) and AGO2 *in vitro*. Lane 1: 22 nt biotin-ssRNA; 2: pre-miRNA; 3: pre-miRNA with Dicer; 4: pre-miRNA with Dicer and 200 ng AGO2; 5: pre-miRNA with Dicer and 500 ng AGO2. Full-length blots are in Supplementary information. (**h**) The effects of AGO2 on the stabilities of mature miR-130b in H1299 cells. H1299 cells were transfected with AGO2 siRNA or miR-138 mimic for 24 h. Cells were co-transfected with an *AGO2* expression plasmid (pCMV-AGO2) for complementation analysis. Cells were then treated with actinomycin D for another 24 h. miRNA expression was determined with qRT-PCR. U6 served as an internal control. miR-130b precursors were prepared by *in vitro* transcription. *P < 0.05 and **P < 0.01 by LSD test. The data are representative of three independent experiments (mean ± s.d.).

In view of the pivotal role of AGO2 in maintaining miRNA abundance, we next explored the biological effects of decreased AGO2 on the expression of miRNAs. Two pairs of miRNA microarrays with total small RNA from H1299 cells treated with AGO2 siRNA or miR-138 mimic and the corresponding control cells were analyzed for miRNA profiling (GEO: GSE69562). Among the differentially expressed miRNAs (Fig. 2a,b), miR-130b decreased the most significantly both in the AGO2 siRNA group and the miR-138 mimic group. A pathway enrichment analysis using miRWalk2.0 database showed that miR-130b was involved in p53 signalling pathway and multiple signaling pathway in NSCLC cells (Supplementary Fig. S1b).

To investigate whether AGO2 could affect the processing of miR-130b, the abundance of pri-miR-130b (primary-miR-130b), pre-miR-130b (precursor-miR-130b), and mature miR-130b were detected using q-PCR in cells with either AGO2 overexpression or AGO2 silencing using AGO2 siRNA or a miR-138 mimic. Forced expression of AGO2 caused a decrease in pre-miR-130b but an increase in miR-130b, while silencing AGO2 expression either with AGO2 siRNA or a regulatory miR138 mimic demonstrated the opposite trend, i.e., an increase in pre-miR-130b but a decrease in miR-130b, with no detectable change in the pri-miR-130b levels (Fig. 2c). These results support the idea that AGO2 is involved in pre-miR-130b to miR-130b processing but not in pri-miR-130b to pre-miR-130b processing. Reconstitution of AGO2 simultaneously with silencing treatment showed a rescue of the silencing effect on both the pre-miR-130b and mature miR-130b abundance to a certain degree (Fig. 2c), demonstrating an AGO2-specific effect. To further test the hypothesis of slicer catalytic activity of AGO2 on pre-miR-130b, *in vitro* cleavage experiments were then performed. The result showed that 60-nt pre-miR-130b could be sliced into the mature miR-130b (21 nt) by Dicer protein as expected but could also be sliced into an unexpected band when treated with AGO2 protein (Fig. 2d). This unexpected intermediate band, named as Ac-pre-miR-130 for AGO2-cleaved pre-miR-130, was recycled from the gel and subjected to sequencing. Sequencing and putative secondary structure results showed that Ac-pre-miR-130b was 47 nt in length and was produced from pre-miR-130b by cleavage of the passenger strand at a position 13 nucleotides from its 5′ end (Fig. 2e), which is different from the reported AGO2 processing of the 3′ arm of pre-miRNAs such as pre-let-7a^27^. In addition, the Ac-pre-miR-130b was further processed by Dicer into mature miR-130b more efficiently than the pre-miR-130b was (Fig. 2f). Indeed, when we co-incubated the pre-miR-130b with recombinant AGO2 and Dicer protein, the production of the intermediate Ac-pre-miR-130 was significantly increased, in a manner dependent on the AGO2 concentration, and thus, the production of mature miR-130b from the 3′ end by Dicer was also elevated (Fig. 2g). Previously, AGO2 processing of pre-let-7a was synchronously performed and was shown to have a different 3′ arm cleavage pattern^27^. Therefore, we demonstrated that AGO2 could facilitate the Dicer-induced processing of miR-130b by production of the intermediate forms of Ac-pre-miR-130b.

Furthermore, we analyzed the effects of AGO2 on the stability of miR-130b. After inhibiting the expression of AGO2 with AGO2 siRNA or miR-138 mimic in H1299 cells, actinomycin D was used to inhibit RNA synthesis. Let-7a and let-7f were chosen as positive controls for AGO2-enhanced stability based on a previous study^28^. In addition, miRNAs that were negatively regulated by AGO2 according to the miRNA microarray results (GEO: GSE69562) were randomly chosen (miR-106b, miR-4478, or miR-19b in this study) as negative controls. Similar to the above results, the abundance of miR-130b was decreased by silencing AGO2 expression, and this decrease was reversed by reconstitution of AGO2 expression in all DMSO cohorts. The effect was further enhanced by actinomycin D inhibition of RNA synthesis. The enhancement was significant in the AGO2-silenced cells (via AGO2 siRNA or miR138 mimic) compared with that of the control small RNA transfection group (Fig. 2h), suggesting an AGO2-specific stability effect in the tested human NSCLC cells.

### Negative regulation of miR-130b by p53 in human NSCLC cells

**Figure 3:**
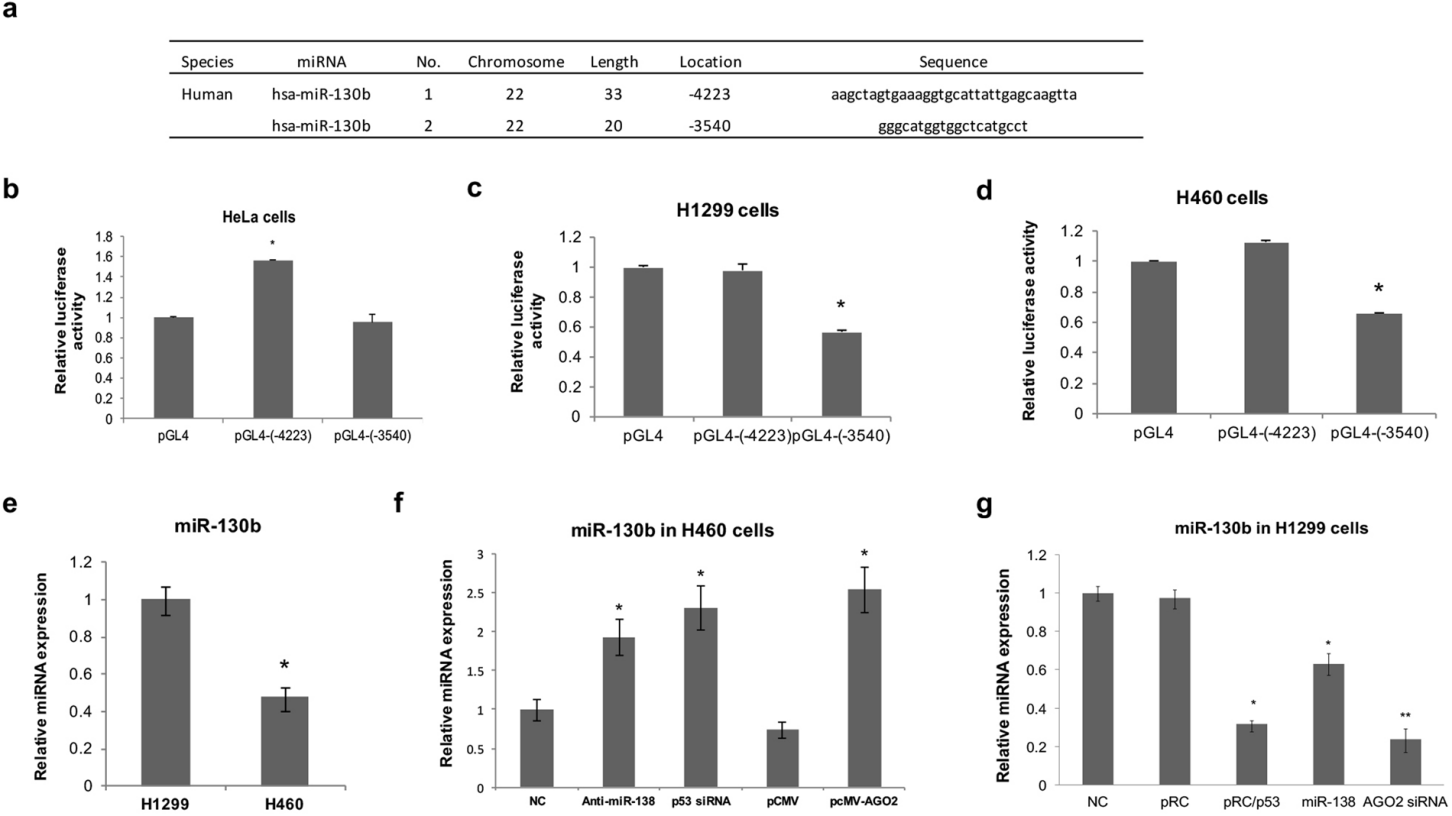
The regulatory effect of p53 on miR-130b. (**a**) Sequences and positions of the predicted p53-binding sites in the promoter of the miR-130b gene. The predicted p53-binding sites were incorporated separately into the basic promoter of a firefly luciferase reporter gene (named pGL4-(-4223) and pGL4-(-3540)). (**b**–**d**) pRC/p53 plasmids were co-transfected with the luciferase reporter plasmid pGL4-(-4223), pGL4-(-3540), or empty pGL4 vector into HeLa (**b**), H1299 (**c**) and H460 (**d**) cells. After 48 h, the luciferase activity was measured. *P < 0.05 *vs*. the control by one-way ANOVA test. (**e**) Quantitative RT-PCR assays were performed to examine the relative miR-130b expression levels in H1299 and H460 cells. *P < 0.05 by rank-sum test. (**f**) Quantitative RT-PCR assays were performed to examine the relative miR-130b expression levels in H460 cells transfected with anti-miR-138, p53 siRNA, or pCMV-AGO2. *P < 0.05 *vs*. NC by one-way ANOVA test. (**g**) Quantitative RT-PCR assays were performed to examine the relative miR-130b expression levels in H1299 cells transfected with pRC/p53, miR-138, or AGO2 siRNA. The qRT–PCR expression values were normalized to that of U6. *P < 0.05 and **P < 0.01 *vs*. the control by one-way ANOVA test. The data are representative of three independent experiments (mean ± s.d.).

Taking into account the results showing that AGO2 regulated miR-130b and the aforementioned p53-miR-138-AGO2 regulatory pathway, we postulated a p53-miR-138-AGO2-miR-130b pathway, which suggested downregulation of miR-130b by p53. We first ruled out the possibility of direct transcriptional upregulation of miR-130b by p53 in NSCLC cell lines by luciferase reporter assay. Luciferase reporter constructs containing two potential binding sites for p53 protein at upstream of miR-130b (locations −4223 and −3540) predicted by BioSun software and a p53MH algorithm^29, 30^ (Fig. 3a), termed pGL4-(−4223) and pGL4-(−3540), respectively, were used to detect whether p53 showed the appropriate transcriptional activity by binding to the predicted sites. After overexpressing p53 in HeLa cells, which have low p53 activity, pGL4-(−4223) showed high luciferase activity compared with the control plasmid, while the luciferase activity of pGL4-(−3540) was unchanged (Fig. 3b). However, overexpression of p53 in H1299 or H460 cells did not enhance the luciferase activity of pGL4-(−4223) or pGL4-(−3540) (Fig. 3c,d). These results suggested that there is no direct transcriptional activation of miR-130b by p53 in human NSCLC cells (H460 and H1299).

We next compared the native expression level of miR-130b in H460 and H1299 cells. The miR-130b content in H1299 cells was significantly higher than that in H460 cells (Fig. 3e). When p53 was downregulated by siRNA in H460 cells, the miR-130b level significantly increased. These results are consistent with the findings obtained by overexpressing *AGO2* and the use of miR-138 inhibitors (anti-miR-138) (Fig. 3f). Similar results were observed in H1299 cells, i.e., overexpression of p53 significantly reduced the miR-130b content, which is consistent with the effects of the miR-138 mimic and AGO2 siRNA in the cells (Fig. 3g). To investigate whether miR-130b was capable of p53 feedback regulation, we also analyzed the possible target sites of miR-130b in p53 using miRNA target prediction software (miRanda 2010, TargetScan or PicTar). We confirmed that there is no target site for miR-130b in p53. Furthermore, the p53 mRNA and protein levels were not significantly changed after transfection of a miR-130b mimic into H460 cells (Supplementary Fig. S2). These results suggested a complex one-way regulatory pathway from p53 to miR-130b via miR-138 and AGO2 in human NSCLC cells.

### Downregulation of miR-130b promotes the expression of *GADD45A* in human NSCLC cells

**Figure 4:**
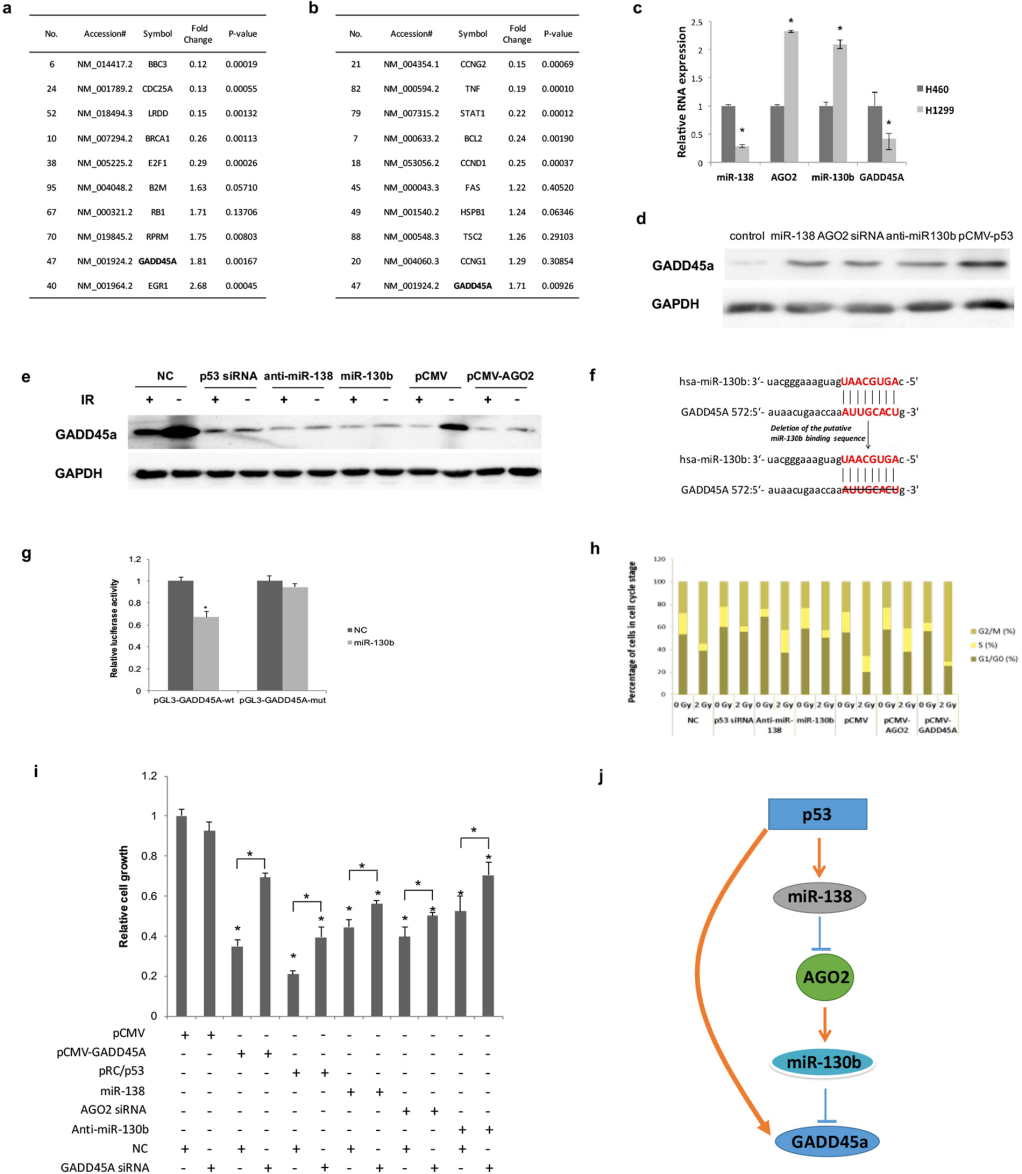
p53-miR-138-AGO2-miR-130b pathway regulation of GADD45A in human NSCLC cells. Human p53 signalling pathway PCR array analysis. p53 signalling pathway gene expression profiles in H1299 cells treated with AGO2 siRNA, miR-138, or negative control were analyzed using a q-PCR array (**a**,**b**). ^a^Only the top ten most significantly changed genes are listed (for more details, see GEO: GSE69561). (**c**) The relative expression levels of GADD45A mRNA, AGO2 mRNA, miR-130b and miR-138 were quantified in H460 and H1299 cells using real-time PCR. GAPDH mRNA and U6 snRNA served as internal controls, separately. *P < 0.05 by rank-sum test. (**d**) GADD45A protein was analyzed with western blotting in H1299 cells transfected with miR-138, AGO2 siRNA, negative control or p53 expression plasmid. Full-length blots are in Supplementary information. (**e**) H460 cells were transfected with p53 siRNA, anti-miR-138, miR-130b, pCMV-AGO2, or control plasmid. GADD45A protein was analyzed with western blotting when cells were treated with or without 2 Gy of IR, and then cultured for 8 hours. Full-length blots are in Supplementary information. (**f**) Schematic representation of the miR-130b target in the 3′ UTR of human *GADD45A* (top). The positions of the miR-138 binding sites correspond to the location of the GenBank accession number NM_001199742. The mutant 3′ UTR without the miR-130b binding sites is shown in the bottom image. (**g**) Dual-luciferase reporter assays were performed to test the interaction between miR-130b and the predicted wild-type *GADD45A* 3′ UTR targeting sequences (pGL3-GADD45A-wt) and mutated targeting sequences (pGL3-GADD45A-mut). *P < 0.05 by rank-sum test. (**h**) H460 cells transfected with p53 siRNA, anti-miR-138, miR-130b, pCMV-AGO2, or control plasmid were treated with 2 Gy of IR. After 24 h, the cells were stained with Hoechst 33342, and the percentage of cells in G2/M, S, and G0/G1 phase was quantified using an IN Cell Analyzer 2000. (**i**) The relative cell growth of H1299 cells. *P < 0.05 by LSD test. The data are representative of at least three independent experiments (means ± s.d.). (**j**) Schematic model indicating a proposed branch pathway in the p53 regulation of GADD45A by which p53 activates miR-138 to downregulate AGO2 and miR-130b.

To determine the biological functions of miR-130b regulation by the p53-miR-138-AGO2 pathway, we inhibited the expression of miR-130b using a miR-138 mimic or *AGO2* siRNA in human NSCLC cells. Two pools of total mRNA from treated cells were subjected to a “Human p53 Signaling Pathway PCR Array” (GEO: GSE69561). Among the 92 genes associated with p53, *GADD45A* was the differentially expressed gene, both in the miR-138-treated group and the AGO2 siRNA-treated group (Fig. 4a,b). In this respect, it appears unique. Subsequent q-PCR indicated that the expression level of miR-130b was negatively correlated to that of *GADD45A* in the tested H460 and H1299 cells (Fig. 4c). miR-130b inhibitors significantly increased the expression levels of the GADD45A protein in H1299 cells, which was consistent with the results observed when p53 and miR-138 were overexpressed or when AGO2 was reduced (Fig. 4d). As wild-type (wt) p53 upregulates *GADD45A* expression after being activated by IR stimulation, we applied ionizing radiation (2 Gy) to H460 cells^10^. The results showed that the expression of *GADD45A* was significantly upregulated in IR-treated H460 cells and decreased after treatment with miR-130b mimic and by p53 siRNA. These observations are consistent with the results obtained by inhibiting miR-138 activity and overexpressing *AGO2* (Fig. 4e). Thus, the results revealed the possibility that miR-138 and miR-130b are involved in the p53-mediated regulation of the expression levels of *GADD45A* in human NSCLC cells. To identify whether *GADD45A* is a target of miR-130b, a miRNA target prediction analysis (http://www.targetscan.org/; http://www.microrna.org/) was performed, and we found potential miR-130b binding sites in the 3′ UTR of *GADD45A* mRNA (Fig. 4f). The full length sequence (641nt, NM_001199742), or the sequence with a deletion mutation of the miR-130b binding sites of the *GADD45A* 3′ UTR was cloned into the downstream region of a firefly luciferase reporter gene (named pGL3-GADD45A-wt and pGL3-GADD45A-mut, respectively). We then assayed the luciferase activity in H1299 cells. The results showed that miR-130b significantly reduced the luciferase activity of the vector pGL3-GADD45A-wt, but not that of the pGL3-GADD45A-mut vector, indicating that miR-130b directly targets the 3′ UTR of *GADD45A* (Fig. 4g).

In trying to address the biological function of the miRNAs in the p53-mediated regulation of GADD45A, we performed a cell cycle assay and a proliferation assay as reported. We first examined the cell cycle changes in H460 cells treated with 2 Gy IR, which induced upregulation of *GADD45A* expression in H460 cells. The results showed that the cell cycle was arrested at G2/M phase (Fig. 4h). We then observed that cell cycle arrest could be reversed by p53 siRNA, a miR-138 inhibitor, or overexpression of *AGO2* or miR-130b. These results mirrored those observed in the *GADD45A* siRNA-treated positive control group. These findings are also consistent with a previous report that GADD45A-induced G2/M phase arrest under IR usually occurs in a p53-dependent manner^31^. In addition, we also explored the effect of *GADD45A* overexpression on the proliferation of H1299 cells. Cell proliferation was significantly inhibited by overexpression of *GADD45A*, which was consistent with the results of p53 overexpression, transfection with the miR-138 mimic and AGO2 siRNA, and treatment with miR-130b inhibitors (Fig. 4i).

As shown in Fig. 4j, we found that p53 could regulate *GADD45A* through an alternative miRNA pathway, which was different from its transcriptional regulation of *GADD45A* as previously reported^14, 15^.

### The p53-miR-138-AGO2-miR-130b pathway regulation of GADD45A differs among species

Since we previously found differences in the regulation of GADD45A via the p53-miR-138 pathway between humans, mice, and rats, we further investigated the existence of species differences in the p53-miR-138-AGO2-miR-130b pathway. The miRNA target prediction analysis suggested that the *AGO2* 3′ UTRs of humans, mice and rats have target sites for miR-138 (Supplementary Fig. S3a) and the *GADD45A* 3′ UTRs of humans, mice and rats contain the target sites for miR-130b (Supplementary Fig. S3b). When we inhibited the expression of p53 with siRNA in H460, NIH3T3 and H9C2 cells, the *GADD45A* mRNA contents in all three cell lines decreased significantly (Supplementary Fig. S3c). The downregulated mRNA level of *GADD45A* was partially rescued by the miR-138 mimic, AGO2 siRNA, or miR-130b inhibitor in human H460 cells. However, only the miR-130b inhibitor had such an effect in mouse NIH/3T3 and rat H9C2 cells (Supplementary Fig. S3c), suggesting that the p53-miR-138-AGO2-miR-130b pathway was absent in mice or rats. Our results revealed that deviation may exist in the p53-miR-138-AGO2-miR-130b regulation pathway of GADD45A among humans, mice and rats.

As all the aforementioned adopted cells are human NSCLC cells, we also used the human hepatoma cell line Hep3B, with a p53 deletion, to test the existence of the p53-miR-138-AGO2-miR-130b-GADD45A pathway. After overexpression of p53 in Hep3B cells, the q-PCR results indicated that the expression levels of miR-138 and *GADD45A* were upregulated, and the expression levels of *AGO2* and miR-130b were downregulated (Supplementary Fig. S4). These results were consistent with those obtained in H460 and H1299 cells, suggesting that the p53-miR-138-AGO2-miR-130b-GADD45A pathway may be universal in its regulation of human cancer development. These observations require further detailed investigation.

## Discussion

It has recently been reported that Ago2, also known as eIF2C2, is important both for maintaining miRNA abundance through its involvement in miRNA processing^32–34^ and enhancement of miRNA stability^28, 35^, and for enhancing miRNA function via assembly of RISC (RNA-induced silencing complex)^36, 37^. A representative example of the intrinsic endonucleolytic activity of Ago2 is the Dicer-independent processing pathway of the conserved vertebrate miRNA miR-451 in which Ago2 directly processes the primary precursor to the mature miRNA^34^. Frequently, Ago2 can cleave pre-miRNA into an additional intermediate form of the nicked hairpin, designated Ac-pre-miRNAs (AGO2-cleaved precursor miRNA), which may serve as a Dicer substrate to be trimmed into miRNAs. The Ac-pre-miRNA was generated by slicing the 3′ arm of pre-miRNAs, which all belong to the 5′-end miRNAs, such as pre-let-7^27^. In this study, we demonstrated that AGO2 is capable of slicing pre-miR-130b into an Ac-pre-miRNA precursor, followed by Dicer-mediated cleavage of intermediates to produce mature miRNAs. However, miR-130b is a 3′-end miRNA, and we demonstrated that pre-miR130b is sliced at the passenger strand from the 5′ end. We show for the first time that a 3′-end miRNA derived from the intermediate precursor Ac-pre-miRNA. Furthermore, the results showed that generation of Ac-pre-miR130b promoted the maturation of miR-130b by Dicer. Moreover, Ago2 increased miR-130b levels by modulating miRNA stability in a slicing-independent manner. The results suggested the pivotal impacts of AGO2 on the abundance of miR-130b and unveiled variant mechanisms of AGO2-mediated processing.

microRNAs are small single-stranded RNA molecules that regulate gene expression in diverse physiological processes. To date, several miRNAs have been identified as p53 target genes that mediate p53-induced signalling pathways^20, 21^. Moreover, p53 is also able to modulate miRNA functions by interfering with Drosha processing machinery, a transcriptionally independent pathway^22^. Because miRNAs are present in the p53 network, p53 functions are thought to be extremely complicated. In this study, we explored whether miRNAs have potential roles in p53 regulation of GADD45A protein. We previously found that human miR-138 was a target gene of p53 in human NSCLC cells^23^. Here, for the first time, we demonstrated that miR-138 downregulated AGO2 expression induced by p53, resulting in decreased miR-130b abundance, which increased GADD45A expression. Our work herein re-confirmed the p53-mediated regulation of GADD45A by demonstrating a miRNA-directed post-transcriptional pathway. We also suggested the p53-miR-138-AGO2 pathway as one mechanism underlying the high expression of AGO2 in a variety of highly invasive tumour tissues with silenced or mutant p53 expression, including myeloma, colon cancer, and liver cancer^38–41^.

A previous study showed that p53 can promote the transcription of miR-130b in human endometrial cancer cells (HEC-50 and HEC-1)^42^, and these data conflict with our findings. We deduced that the direct transcriptional regulatory effect of p53 on miR-130b might be cell-type specific because we reproduced it in Hela cells but not in NSCLC cells. We also deduced that deviation may exist in the p53-miR-138-AGO2-miR-130b regulation pathway of GADD45A among humans, mice and rats, which may be due to differences in the regulation of miR-138 by p53.

In summary, this study provides direct evidence that there is an alternative microRNA-mediated pathway in p53 regulation of GADD45A in human NSCLC cells, in addition to canonical transcriptional regulation. Due to the participation of miRNAs, the mechanisms of p53 regulation of GADD45A are enriched and much more elaborate. Our results also demonstrated the complexity of the miRNA-protein mutual interaction network in the regulation of gene expression in human cells.

## Methods

### Cell culture

The cell lines were obtained from the Cell Resource Center of the Institute of Basic Medical Sciences (Beijing, China), unless otherwise mentioned. The human NSCLC cell lines H460 and H1299 were obtained from Professor Guo Ning (Department of Pathophysiology, Institute of Basic Medical Sciences, Beijing, China), and routinely grown in RPMI-1640 medium (Sigma-Aldrich, Poole, UK), supplemented with 10% foetal bovine serum (FBS). The human hepatoma cell line, Hep3B, was cultured in minimal essential medium (MEM) supplemented with 10% FBS. The mouse embryo fibroblast cell line NIH/3T3 and the rat embryo fibroblast cell line H9C2 were cultured in Dulbecco’s modified Eagle’s medium with 10% FBS.

### Cell transfection and treatment

Cells at 70% confluence were transfected with vectors, siRNAs, miRNA mimics or anti-miRNA (miRNA inhibitor, 2’-O-methyl and modified phosphorothioates), using Lipofectamine 2000 (Invitrogen, Carlsbad, CA, USA) according to the manufacturer’s recommendations. The transcription inhibitor actinomycin D (10 µg/ml in DMSO) was added 24 hours after transfection.

The miRNA mimics and siRNA sequences were as follows: miR-138 mimic, 5′-AGCUGGUGUUGUGAAUCAGGCCG-3′ (sense); miR-130b mimic, 5′-CAGUG CAAUGAUGAAAGGGCAU-3′ (sense); p53 siRNA, 5′-UAUGAAUCGUCGUC CUAUUC-3′ (sense); AGO2 siRNA, GCCUGUAUCAAGCUAGAAA; GADD45A siRNA, ACAUCCUGCGCGUCAGCAAC.

Cell irradiation was performed during the exponential phase of cell growth, with 2 Gy γ-rays from a Co-60 source at a dose rate of 20 Gy/h.

### Western blot analysis

Whole-cell lysates were obtained using the ProteoJET Mammalian Cell Lysis Reagent (Thermo Scientific, Rockford, IL, USA). Proteins (50 μg) were separated on 10% SDS-PAGE gels and transferred to nitrocellulose membranes. The membranes were blocked with 5% non-fat dried milk in TBST (50 mM Tris (pH 7.5), 200 mM NaCl, 0.05% Tween 20) at room temperature for 1 h. The membranes were then incubated with the primary antibody in the blocking solution for 1 h at room temperature, washed three times with TBST for 15 min, incubated with the HRP-conjugated secondary antibody at room temperature for 1 h and then washed three times with TBST. Antigen-antibody complexes were detected using the SuperSignal detection reagents (Thermo Scientific, Rockford, IL, USA). The following antibodies were used: rabbit monoclonal anti-p53, rabbit monoclonal anti-AGO2, rabbit monoclonal anti-GADD45A, mouse monoclonal anti-GAPDH (Cell Signalling Technology, MA, USA) and horseradish peroxidase-conjugated goat-anti-rabbit and goat-anti-mouse secondary antibodies (Santa Cruz. CA, USA).

### Plasmids

The following protein expression plasmids were used: pRC/CMV was used as a negative control (Promega, Madison, WI, USA), pRC/p53 containing the entire p53 coding region was obtained from Sino Biological (Sino Biological Inc, Beijing, China), and pCMV/AGO2 and pCMV/GADD45A were purchased from OriGene (OriGene Technologies, Rockville, MD, USA). The reporter plasmids used for the transcriptional activation assay were as follows: pGL4.26[luc2/minP/Hygro] (pGL4 for short) was designed for high expression and reduced anomalous transcription (Promega). The p53-binding site oligonucleotides (bold) containing the *Xho*I site at the 5′-end and the *Hin*dIII sequence (italic) at the 3′-end (miR-130b (−4223): 5′-*CTCGAG* **AAGCTAGTGAAAGGTGCATTATTGAGCAAGTTA** *AAGCTT*-3′ and miR-130b (−3540): 5′-*CTCGAG* **GGGCATGGTGGCTCATGCCT** *AAGCTT*-3′) were separately inserted upstream of a minimal promoter of the luc2 gene and named pGL4-(−4223) and pGL4-(−3540), respectively. The following reporter plasmids were used for the miRNA target assay: almost the entire full length sequence of the human AGO2 3′ UTR (879 nt, NM_001164623) and the GADD45A 3′ UTR (641 nt, NM_001199742) were PCR amplified using genomic DNA from H460 cells. The final PCR product was cloned into a pGL3-Control Vector (Promega) between *Mlu*I and *Bgl*II restriction enzyme sites located downstream of the firefly luciferase reporter gene. These constructs were named pGL3-AGO2-Full and pGL3-GADD45A-wt. The wild type (WT) AGO2 3′UTRs with deletion of one or two of the predicted miR-138 binding sites were cloned into pGL3-Control vector and named pGL3-AGO2-Mut1, pGL3-AGO2-Mut2 or pGL3-AGO2-Mut-all. The WT GADD45A 3′UTR lacking the predicted miR-130b binding site was cloned into a pGL3-Control Vector and named pGL3-GADD45A-mut. All the miRNA target binding sites were mutated using a MutanBEST mutation kit (Takara, Tokyo, Japan). The primers used for cloning (bold italics for restriction sites) are listed in Table S1.

### Dual-luciferase reporter assay

Reporter plasmids containing the WT or mutated 3′UTRs and control plasmid [cytomegalovirus (CMV)–driven Renilla luciferase construct, pRL-CMV] (Promega), and miRNA mimics or negative control mimics were co-transfected into cells using Lipofectamine 2000 (Invitrogen), according to the manufacturer’s instructions. After 48 h, the reporter activity was measured using a dual-luciferase reporter gene assay kit (Promega).

### Growth rate assay

Relative cell growth was assessed indirectly by an MTT (3-(4,5- dimethylthiazol-2-yl)-2,5-diphenyltetrazolium bromide) assay. H1299 cells (1 × 10^4^) were seeded into 100 μl of medium per well in 96-well plates and incubated (37 °C, 5% CO_2_) overnight. After treatment, 10 μl of MTT solution (5 mg/ml) was added to each well and incubated for 4 h. The culture medium was then removed, the wells were dried and formazan was resolved with 100 μl/well dimethyl sulphoxide for 30 min. The optical density was measured at 560 nm and the background absorbance at 650 nm was subtracted. Relative cell growth was expressed as the percentage of the optical density vs. that of the control group.

### Quantitative real-time PCR

Total RNA was isolated using the TRIzol reagent (Invitrogen). Relative mRNA and pri-miRNA levels were determined with qRT-PCR using a Stratagene MX3000 P system, One-Step SYBR PrimeScript RT-PCR Kit (Takara), and specific primers. Cycle threshold (CT) values were determined using MX3000p software (version 4.10) with amplification-based threshold determination and adaptive baseline analysis options. GAPDH was used as the endogenous control.

miRNAs and pre-miRNAs were quantified using an All-in-One miRNA qRT-PCR Detection Kit (GeneCopoeia, Rockville, MD, USA). For single-step cDNA synthesis, poly(A) polymerase was used to add poly(A) tails to the 3′ end of miRNAs, and M-MLV reverse transcriptase and a unique oligo(dT) adaptor primer were used to reverse transcribe poly(A) miRNAs. An All-in-One q-PCR Mix containing SYBR Green was used to detect specific reverse-transcribed miRNAs using a Universal Adaptor PCR primer and a miRNA-specific primer. miRNAs were normalized against U6 snRNA. The results are expressed in arbitrary units and are representative of three independent experiments. Primers for the quantitative real-time PCR sequences are shown in Table S2.

### Preparation of pre-miRNA substrates

The pre-miRNAs used in this study were generated by *in vitro* transcription using a TranscriptAid T7 High Yield Transcription Kit (Thermo Scientific, Rockford, IL, USA), according to the manufacturer’s instructions. The double-stranded DNA templates with a T7 RNA polymerase promoter sequence were synthesized by Invitrogen: pre-mir-130b sense, 5′-taatacgactcactatagACTCTTTCCCTGTTGCAC TACTATAGGCCGCTGGGAAGCAGTGCAATGATGAAAGGGCAT-3′; pre-let-7a-3 sense, 5′-taatacgactcactatagTGAGGTAGTAGGTTGTATAGTTTGGGG CTCTGCCCTGCTATGGGATAACTATACAATC-3′; and Ac-pre-miR-130b sense, 5′-taatacgactcactatagUGCACTACTATAGGCCGCTGGGAAGCAGTGCAATGATG AAAGGGCAT-3′; Ac-pre-let-7a-3 sense: 5′-taatacgactcactatagTGAGGTAGTAGGT TGTATAGTTTGG GGCTCTGCCCTGCT ATGGGATAACTATACAATC-3′.

### Pre-miRNA substrate cleavage assays and northern blotting

For *in vitro* pre-miRNA processing assays, commercial recombinant Dicer enzyme (Takara) and AGO2 protein (OriGene Technologies) were used. The Dicer enzyme (2 U) or AGO2 protein (200 ng) was incubated with 20 pmol of RNA substrates in 1× reaction buffer (150 mM NaCl, 20 mM Tris-HCl (pH 8.5), 2.5 mM MgCl_2_) and 2 units of RNase inhibitor (Thermo Scientific). These mixtures were incubated at 37 °C for 4 h. The products were purified by phenol-chloroform extraction, followed by sodium acetate-ethanol precipitation at −20 °C.

The reaction products were separated on 7 M urea-denaturing 15% polyacrylamide gels, and then blotted onto Hybond-N + membranes (GE Healthcare) using a Trans-Blot SD Semi-Dry Transfer Cell (Bio-Rad, Berkeley, CA, USA). Hybridization was performed in Perfect Hyb solution (TOYOBO, Osaka, Japan) containing 1 ng/ml of each biotin-labelled probe (Invitrogen) for 14 h. The membranes were washed in 2× SSC with 0.1% SDS, and the signals were detected using a Chemiluminescent Nucleic Acid Detection Module (Thermo Scientific) according to the manufacturer’s protocol. The probes labelled with biotin in this study were as follows: anti-miR-130b probe (5′-bio-TTGCCCTTTCTTCTTTGCTCTG-3′) and anti-let-7a-3 probe (5′-bio-TTCTTTTC TTCCTTCTTCCTCT-3′).

### Cloning of cleavage products

The processed RNAs were separated on 7 M urea-denaturing 15% polyacrylamide gels, and the gel was stained with SYBR Gold (Invitrogen). The indicated band was excised from the gel and purified. The purified RNA was cloned using a Small RNA cloning kit (Takara) and sequenced.

### Cell cycle analysis

H460 cells transfected with p53 siRNA, anti-miR-138, miR-130b, pCMV-AGO2 or control plasmid were treated with or without 2 Gy IR. After 24 h, the cells were labelled for 45 min with 5 µM Hoechst 33342 and imaged with an IN Cell Analyzer 2000. DNA cell cycle profiles are shown as Hoechst 33342 total intensity per nucleus (density × area) plotted using Spotfire™ DecisionSite™ as a binned histogram on the X-axis, with a count of nuclei per data bin on the Y-axis. Each histogram was derived from IN Cell Investigator analysis of a single image field from four replicate wells per treatment, imaged with a 10 × 0.45 NA objective.

### Microarray and q-PCR array analysis

miRNA microarray assays were performed by a service provider (LC Sciences, Houston, USA). For the microRNA microarray assay, a sample of total cellular RNA (2–5 µg) was size fractionated using a YM-100 Microcon centrifugal filter (GE Healthcare) and isolated small RNAs (<300 nt) were 3′-extended with a poly(A) tail using poly(A) polymerase (New England Biolabs, Peabody, NB, USA). An oligonucleotide tag was ligated to the poly(A) tail for fluorescent staining, and different fluorescent tags were used for each of two RNA samples in dual-sample experiments. Hybridization was performed overnight on a Paraflo microfluidic chip using a microcirculation pump (Atactic Technologies, Houston, TX, USA). After hybridization, fluorescence labelling was detected using tag-specific Cy3 and Cy5 dyes. Hybridization images were collected using a laser scanner (GenePix 4000B, Molecular Devices, Wokingham, West Berkshire, UK) and digitized using an Array-Pro Analyzer v4.5 (Media Cybernetics, Rockville, MD, USA). A low standard threshold was set as a fold difference (log2 ratio) <−0.3 (downregulated) or >0.3 (upregulated) with P-values of <0.05 to show all possible differential miRNAs signals.

Transcription Array Analyses were performed with Affymetrix Human Transcriptome Array 2.0 (Affymetrix, Santa Clara, CA, US). Total RNA (200 ng) from each of the experimental and control group samples was amplified, labelled and purified using an Affymetrix WT Amplification Kit (Cat# 902224, Affymetrix) and GeneChip WT Terminal Labeling Kit (Cat#900671, Affymetrix). We followed the manufacturer’s instructions to obtain biotin-labelled cDNA. Array hybridization and washing were performed using GeneChip® Hybridization, Wash and Stain Kit (Cat#900720, Affymetrix) in a Hybridization Oven 645 (Cat#00-0331-220 V, Affymetrix) and Fluidics Station 450 (Cat#00-0079, Affymetrix) as recommended by the manufacturer’s instructions. Slides were scanned with a GeneChip® Scanner 3000 (Cat#00-00212, Affymetrix, Santa Clara, CA, US) and Command Console Software 3.1 (Affymetrix) with default settings. Raw data were normalized using Expression Console software. The results were considered statistically significant for unadjusted P-values < 0.05 and fold-changes >1.5.

Quantitative detection of the expression of multiple genes involved in the p53 pathways was performed using a human p53 signalling q-PCR primer array (Cat#HAQPA-133-01, GeneCopoeia). Two µg of RNA was used for the cDNA synthesis reaction. The PCR program was performed at 95 °C for 10 minutes, followed by 40 cycles of 95 °C for 15 seconds and 60 °C for 1 minute. Each experiment was performed in triplicate. We analyzed the q-PCR results with GeneCopoeia’s online Data Analysis System (www.genecopoeia.com.cn/product/qpcr/analyse/). The relative expression was calculated using the comparative Ct method. Array genes were normalized to the average value of the housekeeping genes B2M, RPL13A, GAPDH, and ACTB.

### Statistical analysis

All data were processed using SPSS software, version 10.0, and are expressed as the mean ± SD. The data were subjected to a Shapiro-Wilk (W) test for normality. For non-normally distributed data, a nonparametric Wilcoxon signed rank test was used to evaluate the statistical differences between groups. When the data were approximately normally distributed, a comparison of data from multiple groups was performed using one-way ANOVA, and multiple comparisons of means were analyzed by the LSD method. Groups with values of P ≤ 0.05 were considered to be statistically significant.

### Accession numbers

The microarray and q-PCR array data are available in the Gene Expression Omnibus database under the accession numbers GSE69482, GSE69561 and GSE69562.

## Electronic supplementary material

Supplementary Information
