# Supplementary material for: An alternative microRNA-mediated post-transcriptional regulation of GADD45A by p53 in human non-small-cell lung cancer cells
Source: Sci Rep. 2017 Aug 2;7:7153. doi: 10.1038/s41598-017-07332-3 (PMC5541050; doi:10.1038/s41598-017-07332-3)

An alternative microRNA-mediated post-transcriptional regulation of GADD45A by p53 in human non-small-cell lung cancer cells

Jie Li[1]*, Jie Dong[1]*, Shaohua Li[1]*, Wei Xia[1], Xueting Su[1], Xingliang Qin[1], Ying Chen[1], Hongmei Ding[1], Hui Li[1], Aixue Huang[1], Chenjun Bai[1], Tongnan Hu[2], Chenglong Wang[2], Bingfeng Chu[2†], Ningsheng Shao[1†]

SUPPLEMENTARY INFORMATION

Contains supplementary figures (S1 to S4), supplementary tables (S1, S2) and full unedited figures.

Supplementary Figures

a

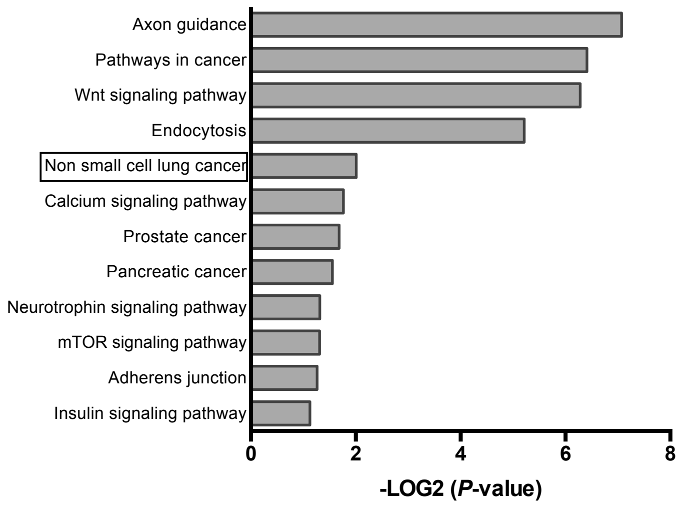

b

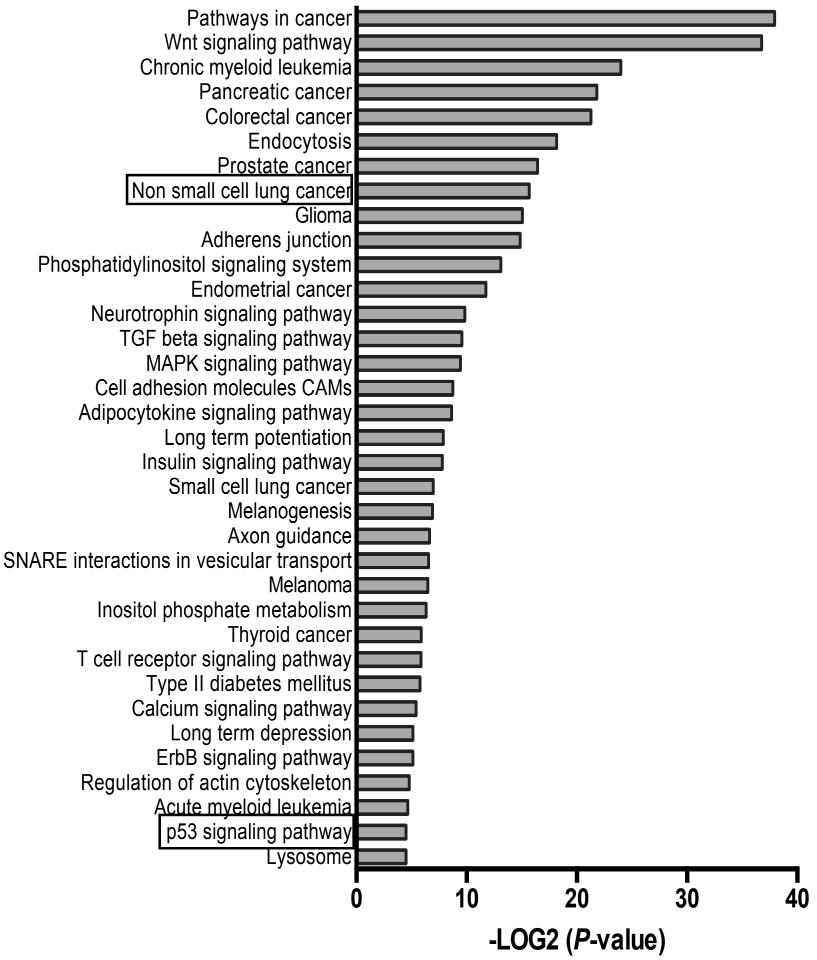

Figure S1. Pathway enrichment analysis of miR-138 (a) and miR-130b (b).

a

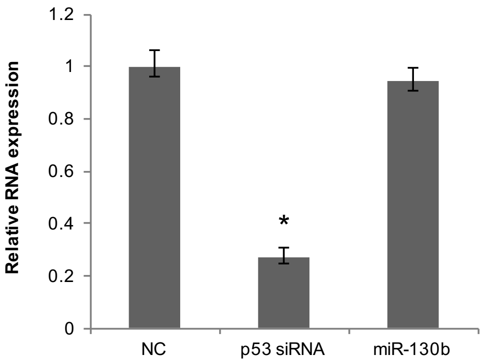

b

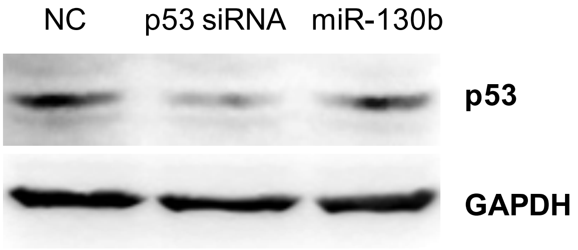

Figure S2. MiR-130b has no effect on p53. H460 cells transfected with p53 siRNA, miR-130b or negative control for 48h. (**a**) p53 mRNA was quantified by real-time PCR. *GAPDH* served as an internal control. *P<0.05 *vs*. NC. (**b**) p53 protein levels analysed by western blotting.

a

```
                    miR-138/Eif2c2 Alignment        mirSVR score:      -0.5756
                                                    PhastCons score:    0.6465
Site1
       3' gccggaCUAAGUGUUGUGGUCGA 5'  hsa-miR-138
                |||||||  |  |||||||
    30:5' gaguggGAUUCACGAGACCAGCU 3'  Human EIF2C2

       3' gccggaCUAAGUGU----UGUGGUCGA 5'  mmu-miR-138
                ||||||||    |  |||||||
    30:5' aaguugGAUUCACACGAGACCAGCU 3'  Mouse EIF2C2

       3' gccggaCUAAGUGU----UGUGGUCGA 5'  rno-miR-138
                ||||||||    |  |||||||
    30:5' aaguugGAUUCACACGAGACCAGCU 3'  Rat EIF2C2

Site2  3' gccGGACUAAGUGUUGU--GGUCGa 5'  hsa-miR-138
            |||    | |     |||  |||||
    75:5' agcCCU----UCCGUGACAGCCAGCa 3'  Human EIF2C2

       3' gccGGACUAAGUGUUGU--GGUCGa 5'  mmu-miR-138
            |||    |       |||  |||||
    77:5' agcCCU----UCUGUGACAGCCAGCa 3'  Mouse EIF2C2

       3' gccGGACUAAGUGUUGU--GGUCGa 5'  rno-miR-138
            |||    |       |||  |||||
    77:5' agcCCU----UCUGUGACAGCCAGCa 3'  Rat EIF2C2
```

b

	predicted consequential pairing of target region	context+
Position 108-114 of Human GADD45A 3' UTR	5' ...AAUAACUGAACCAAAUUGCACUG...	-0.22
	|||||||	
hsa-miR-130b	3' UACGGGAAAGUAGUAACGUGAC	
Position 108-114 of Mouse GADD45A 3' UTR	5' ...AAUAACUGAACCAAAUUGCACUG...	-0.22
	|||||||	
mmu-miR-130b	3' UACGGGAAAGUAGUAACGUGAC	
Position 123-129 of Rat GADD45A 3' UTR	5' ...AAUAACUGAACCAAAUUGCACUG...	-0.22
	|||||||	
rno-miR-130b	3' UACGGGAAAGUAGUAACGUGAC	

c

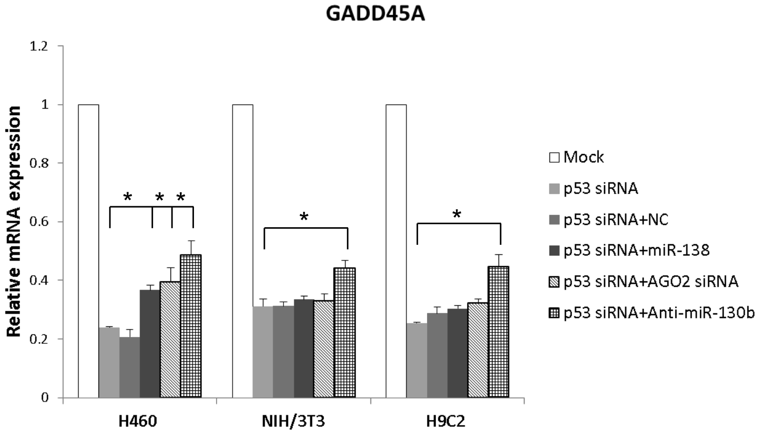

Figure S3. The p53-miR-138-AGO2-miR-130b pathway regulation of GADD45A is different among human, mouse and rat cells. (**a**) *AGO2* mRNA 3′ UTRs of Mouse and rat have similar miR-138 targeting sites to that of human *AGO2*. (**b**) Predicted miR-130b target sequences in mouse and rat *GADD45A* mRNA 3′ UTR by TargetScan were the same as those in human *GADD45A*. (**c**) H460, NIH/3T3 and H9C2 cells were transfected with the indicated siRNAs and miRNAs for 48h. *GADD45A* mRNA in the cells was quantified by real-time PCR . *GAPDH* mRNA served as the internal control. **P*<0.05 *vs*. control. Data are representative of at least three independent experiments (means ± s.d.).

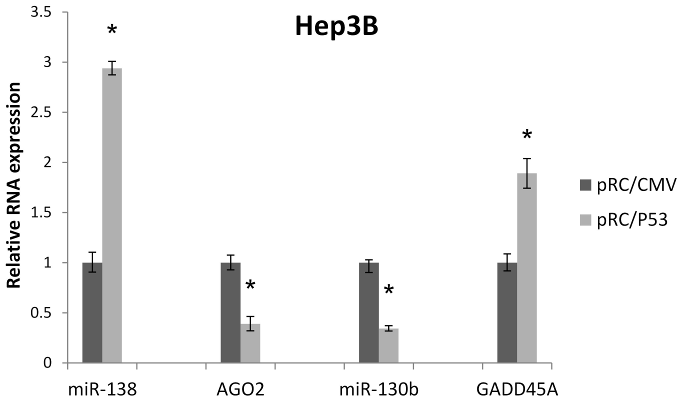

Figure S4. *GADD45A* mRNA, *AGO2* mRNA, miR-130b and miR-138 relative expression levels were quantified by real-time PCR in Hep3B cells (human hepatocellular carcinoma cell). *GAPDH* mRNA and U6 snRNA served as internal controls, separately. *P<0.05 *vs*. control. Data are representative of at least three independent experiments (means ± s.d.).

Supplementary Tables

Table S1. Primers used for plasmids construction

Primer name		Sequence (5'-3'，bold italic for restriction sites)
pGL3-AGO2-Full	Forward	CG***ACGCGT***GTGTTTAGCGATTGTGTACC
	Reverse	TTTATCACCACAGACCCGTA***AGATCT***TCC
pGL3-AGO2-Mut1	Forward	TACACTCAGACCAACAGATGG
	Reverse	CCACTCGGTACACAATCGCT
pGL3-AGO2-Mut2	Forward	CGAACATGAGACGTCATTG
	Reverse	GCTGGCCATCTGTTGGTCTGA
pGL3-AGO2-Mut-all	Forward	CGAACATGAGACGTCATTG
	Reverse	CCACTCGGTACACAATCGCT
pGL3-GADD45A-wt	Forward	CG***ACGCGT***GTCAACTTATTTGTTTTTGCCGG
	Reverse	GAAATGATGCAATTATTCATACCAG***AGATCT***TCC
pGL3-GADD45A-mut	Forward	CCTTTGTAGTTACTCAAGC
	Reverse	TTCAGATGCCATCACCGTTC

Table S2. Primers for quantitative real-time PCR

Name		Sequence（5'-3'）
Human p53	Forward	GAGGTTGGCTCTGACTGTACC
	Reverse	TCCGTCCCAGTAGATTACCAC
AGO2	Forward	TCCACCTAGACCCGACTTTGG
	Reverse	GTGTTCCACGATTTCCCTGTT
GADD45A	Forward	GAGAGCAGAAGACCGAAAGGA
	Reverse	CACAACACCACGTTATCGGG
GAPDH	Forward	ACAACTTTGGTATCGTGGAAGG
	Reverse	GCCATCACGCCACAGTTTC
U6	Forward	CTCGCTTCGGCAGCACA
	Reverse	GCGAGCACAGAATTAATACGAC
pri-miR-130b	Forward	CATTCCAGGTCTCAGATCC
	Reverse	CACCTCAACCTTCTCAACT
pre-miR-130b	Forward	CCGACACTCTTTCCCTGTTGCA
	Reverse	TGACCGATGCCCTTTCATCA
miR-138	Forward	GCCGCAGCTGGTGTTGTGAAT
	Reverse	GCGAGCACAGAATTAATACGAC
miR-130b	Forward	CGGCGCAGTGCAATGATGAAA
	Reverse	GCGAGCACAGAATTAATACGAC
let-7a	Forward	CGGCGTGAGGTAGTAGGT
	Reverse	GCGAGCACAGAATTAATACGAC
let-7f	Forward	CGGCGTGAGGTAGTAGAT
	Reverse	GCGAGCACAGAATTAATACGAC
miR-106b	Forward	GCGAGCACAGAATTAATACGAC
	Reverse	GCGAGCACAGAATTAATACGAC
miR-4478	Forward	TGGAG GAGGCTGAGCTGA
	Reverse	GCGAGCACAGAATTAATACGAC
miR-19b	Forward	CGGCGTGTGCAAATCCATGCAA
	Reverse	GCGAGCACAGAATTAATACGAC
Unique oligo(dT) adaptor primer		GCGAGCACAGAATTAATACGACTCACTATAGGTTTTTT TTTTTTTTTTTTVN

Full unedited gel for figure 1e

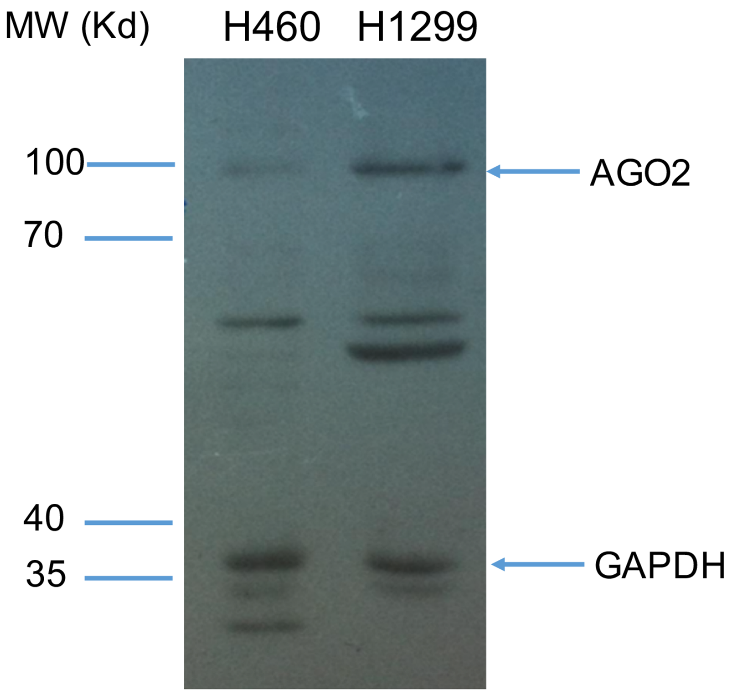

Full unedited gel for figure 1f

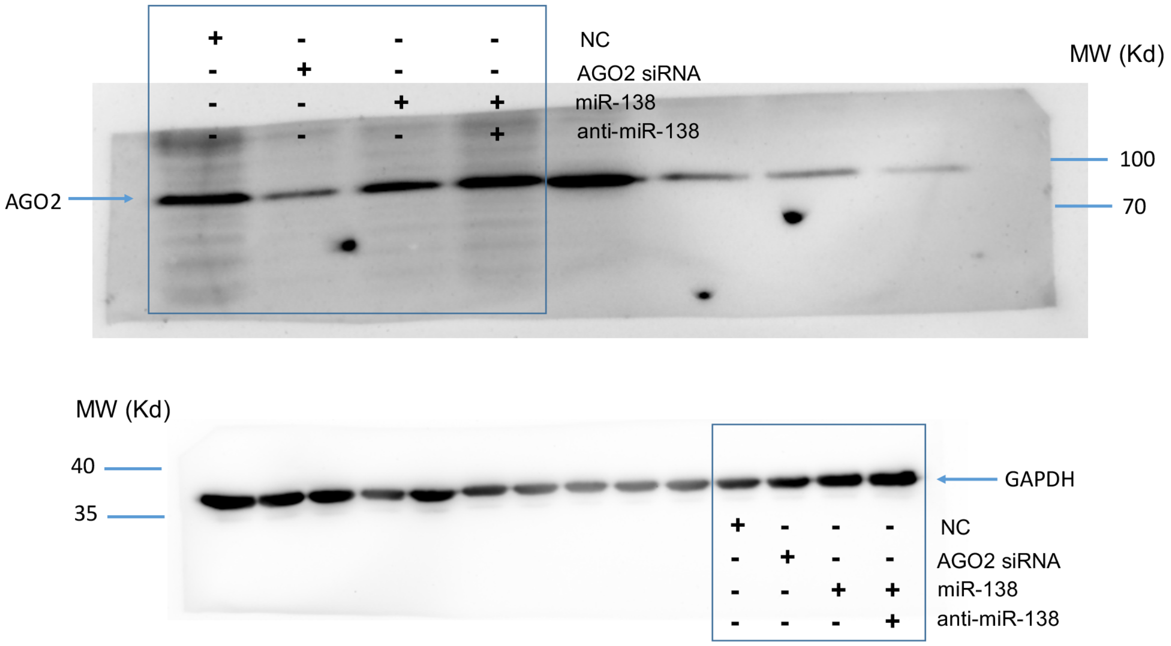

Full unedited gel for figure 1g

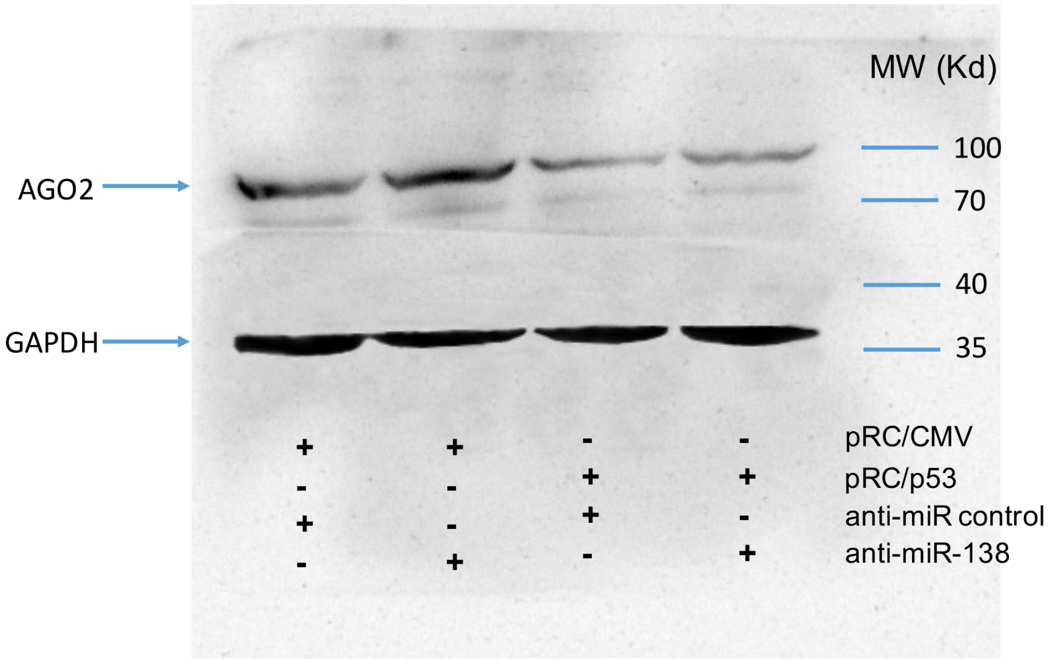

Full unedited gel for figure 1h

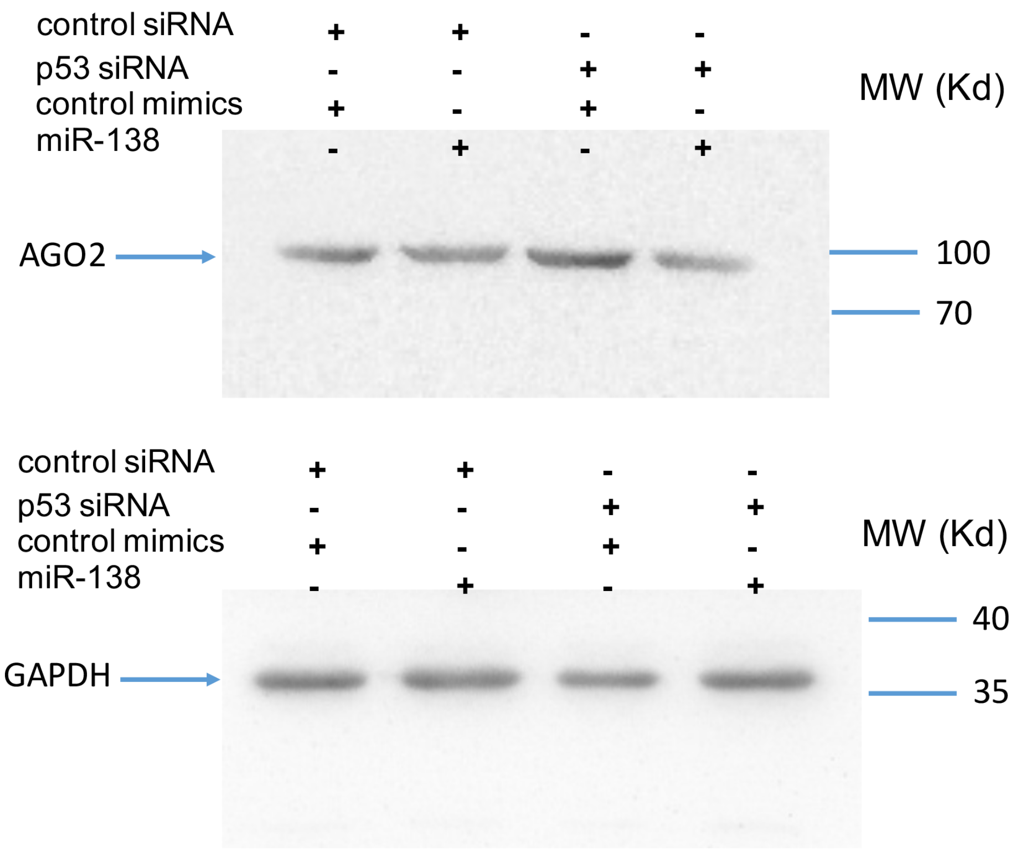

Full unedited gel for figure 2d

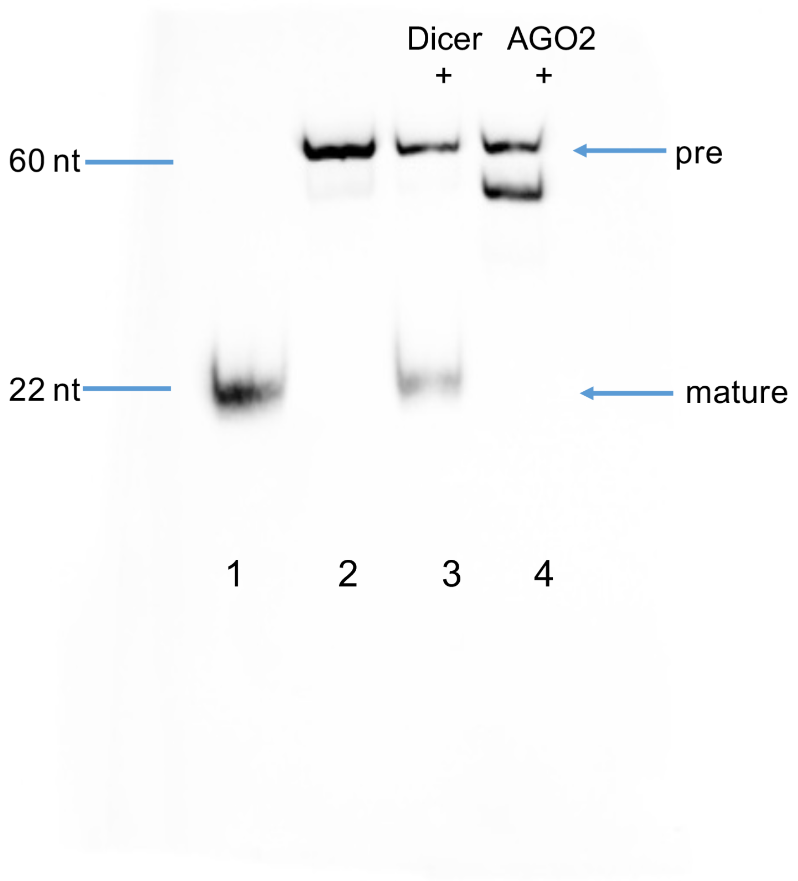

Full unedited gel for figure 2f

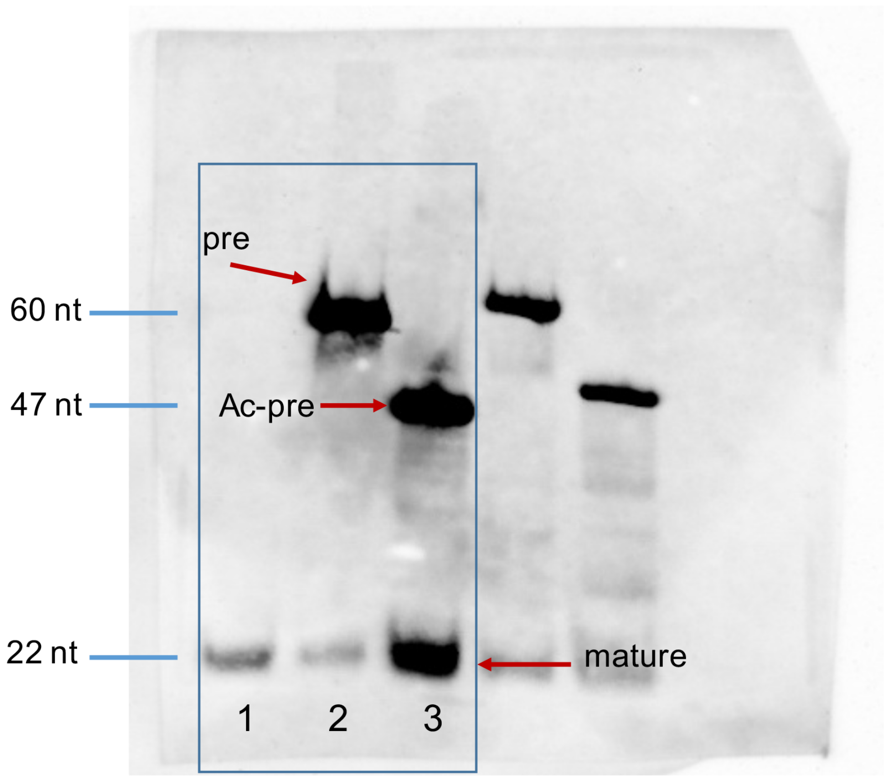

Full unedited gel for figure 2g

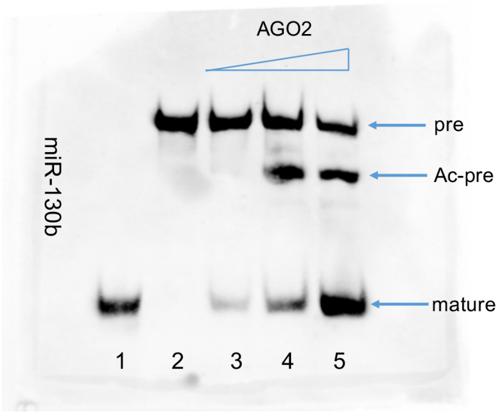

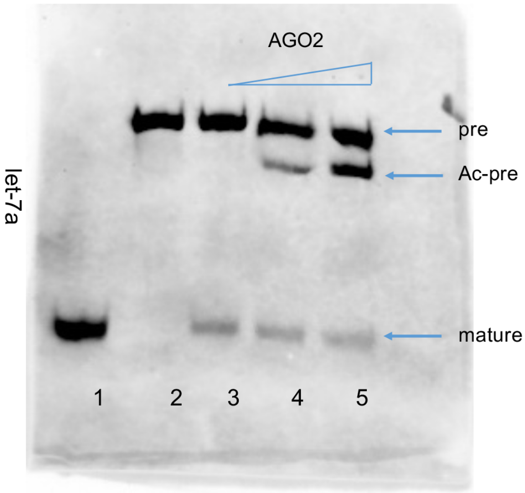

Full unedited gel for figure 4d

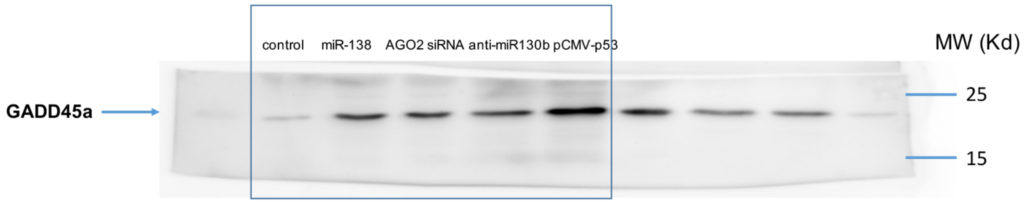

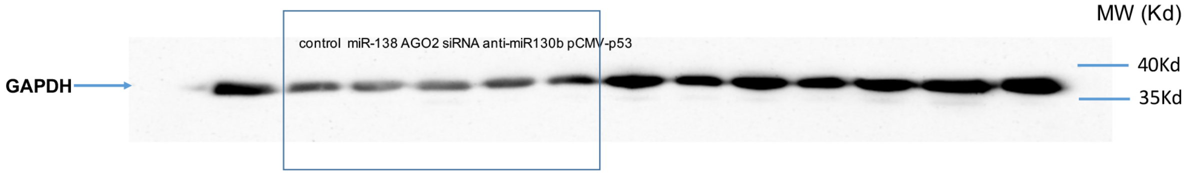

Full unedited gel for figure 4e

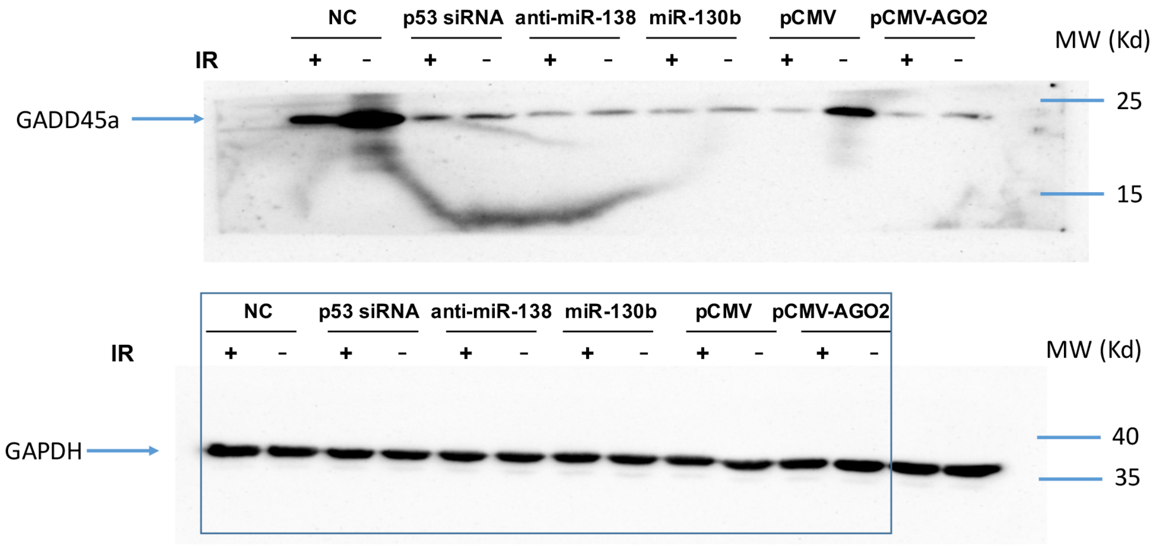

Supplement: Supplementary file 1 — Supplementary Information [file 41598_2017_7332_MOESM1_ESM.pdf]
